# Versatile inhibitory effects of the flavonoid-derived PI3K/Akt inhibitor, LY294002, on ATP-binding cassette transporters that characterize stem cells

**DOI:** 10.1186/2001-1326-1-24

**Published:** 2012-10-13

**Authors:** Yasuo Imai, Hidetsugu Yamagishi, Yuko Ono, Yoshihiko Ueda

**Affiliations:** 1 Department of Pathology, Dokkyo Medical University Koshigaya Hospital, 2-1-50 Minami-Koshigaya, Koshigaya, Saitama, 343-8555, Japan

**Keywords:** ABC transporter, BCRP, P-glycoprotein, MRP1, PI3K/Akt, LY294002, Chemotherapy, Stem cell

## Abstract

Stem cells are undifferentiated cells capable of proliferation, self-renewal, and production of a large number of differentiated progeny. Stem cells exist even in malignancies. They are called cancer stem cells, which may represent the origin of these tumors and may be one of the reasons of chemoresistance. The phosphatidylinositol-3-kinase (PI3K)/Akt pathway is important for the maintenance of pluripotency in stem cells. Flow cytometry assay for identifying stem cells defines a side population of cells that displays low fluorescent dye and is highly enriched for stem cells. The dye efflux is attributed to expression of ATP-binding cassette transporters such as P-glycoprotein and breast cancer resistance protein (BCRP)/ABCG2, which also transport a variety of anticancer drugs. The PI3K/Akt pathway can modulate functions of ABC transporters through various mechanisms. Reportedly, inhibition of the PI3K/Akt pathway caused BCRP translocation in hematopoietic stem cells and glioma stem-like cells. On the other hand, a PI3K inhibitor, LY294002, reversed multidrug resistance in cancer cells that overexpress BCRP not by affecting BCRP translocation but putatively as a competitive inhibitor. Other PI3K inhibitors, wortmannin and PI-103, did not reverse BCRP-mediated drug resistance. Since LY294002 is a derivative of quercetin that is a naturally occurring flavonoid, its chemical structure is similar to those of a group of flavonoids but those of wortmannin and PI-103 are quite different. It is known that many flavonoids are inhibitors of BCRP and PI3K. LY294002 has also been reported to exert inhibitory effects on multidrug resistance-associated protein 1 (MRP1) function via dual mechanisms, competitive block of substrate transport and modulation of expression. Furthermore, LY294002 has been found to antagonize transport activity of P-glycoprotein without influencing its expression. Taken together, LY294002 can inhibit all BCRP, P-glycoprotein, and MRP1, which are three major ABC transporters that are highly expressed in stem cells and cause multidrug resistance. Due to its versatile effects, LY294002 could be a lead compound for developing more effective and tolerable reagents for cancer treatment.

## Introduction

Stem cells are undifferentiated cells capable of proliferation, self-renewal, production of a large number of differentiated progeny, and regeneration of tissues [1]. In addition to embryonic stem (ES) cells, stem cells also exist in various adult tissues such as skin, bone marrow, liver, striated muscle, and brain [1]. After tissue damage, they not only replenish the tissue they originally resided but also may contribute to repair another tissue. Cells with stem-cell qualities have also been identified in malignancies of hematopoietic origin and in some solid tumors [2]. The cells, called cancer stem cells or cancer stem-like cells, share many properties of normal stem cell, such as relative quiescence, resistance to drugs and toxins, an active DNA-repair capacity, and a resistance to apoptosis, thus providing for a long lifespan.

For decades, the unregulated growth of cancers was attributed to the serial acquisition of genetic events that resulted in the activation of oncogenes and silencing of tumor suppressor genes involved in apoptosis. Now, it is recognized that another key event in tumor progression is the alterations of genes involved in regulation of stem cell renewal. When cancer cells of different origins were analyzed for their proliferative potential in various assays, only a small minority of cells were able to proliferate extensively. Those cells, i.e., cancer stem cells, are derived either from transformed normal stem cells or more differentiated progenitor cells that have acquired the ability of self-renewal as a result of oncogenic mutation [3]. Thus, cancer stem cells may represent the origin of cancer and furthermore would play a pivotal role in maintaining cancer cell population. In this point of view, characterization of cancer stem cells would be significant and important for clinical cancer treatment.

Stem cells can be purified based on the efflux of fluorescent dyes such as rhodamine 123 (rho123) and Hoechst 33342 (Hoechst). A widely used flow cytometry assay for identifying stem cells defines a side population (SP) of cells displaying low Hoechst fluorescence and comprising about 0.05% of total cells [4]. This SP population is highly enriched for lineage-specific stem cells. The dye efflux component of the SP phenotype has been assumed to express ATP-binding cassette (ABC) transporters such as P-glycoprotein (P-gp), encoded by the *multidrug resistance 1* (*MDR1*) gene, and breast cancer resistance protein (BCRP)/ABCG2. BCRP expels Hoechst but not rho123, while P-gp expels both Hoechst and rho123. Accordingly, normal and cancer stem cells express high levels of P-gp and BCRP. These two ABC transporters and multidrug resistance-associated protein 1 (MRP1)/ABCC1 constitute the three principle ABC transporters implicated in multidrug resistance. MRP1 has also been found to be expressed in SP cells [4].

### Chemoresistance and cancer stem cells

Cancer cells can acquire resistance to chemotherapy by various mechanisms. Since most cancer cells are genetically unstable at either chromosomal or nucleotide level, cancer cells that recur after initial chemotherapy usually acquire drug resistance by accumulating genetic changes which lead to a selective advantage that allows them to survive the chemotherapy and reconstitute the population of tumor cells. Some of these genetic changes observed in cancer cells grown in tissue culture are a loss of cell surface receptor or transporter for a drug, specific metabolism of a drug, or alteration by mutation of the specific target of a drug. In such cases, resistance to only a small number of related drugs is observed. In addition, cancer cells often express mechanisms that confer simultaneous resistance to many structurally and functionally unrelated drugs. This phenomenon, called multidrug resistance, can result from limitation of cellular drug accumulation by limiting uptake, enhancing efflux or affecting membrane lipids such as ceramid [5]. Blocking apoptosis, activation of detoxifying drugs, repair of DNA damage, and alteration in the cell cycle and checkpoints also render cancer cells resistant to the cytotoxic drugs [6]. Alternatively, cancer stem cells are intrinsically resistant to chemotherapy through their quiescence, their capacity for DNA repair, and expression of ABC transporters such as BCRP, P-gp, and MRP1. Cancer stem cells survive chemotherapy and support regrowth of the tumor. Those ABC transporters transport both hydrophobic and hydrophilic compounds, and play important roles in normal physiology in the transport of drugs [2]. Therefore, modifying functions of the ABC transporters would be one of the attractive strategies for overcoming multidrug resistance of cancer stem cells.

### The PI3K/Akt pathway in cancer stem cells

Large scale transcriptional profiling analysis revealed that many of the transcripts enriched in human ES cells fell within three major signaling categories belonging to the Ras/MAPK/ERK pathway, the phosphatidylinositol-3-kinase (PI3K)/Akt pathway, and NFκB signaling pathways [7]. Among these, the PI3K/Akt pathway had been shown to be important for the maintenance of pluripotency in mouse ES cells [8,9]. LY294002, which is a specific PI3K inhibitor, was shown to cause prominent cell differentiation in human ES cells [7]. PI3K enzymes are normally regulated by growth factors and serve to phosphorylate phospholipids at the plasma membrane. Activated PI3K phosphorylates phosphatidylinositol 4,5-bisphosphate (PIP_2_) and generates phosphatidylinositol 3,4,5-triphosphate (PIP_3_) which acts as a second messenger. Akt interacts with PIP_3_ and subsequently translocates to the plasma membrane. Akt plays a critical role in controlling the balance between survival and apoptosis. Recent studies indicate that numerous components of the PI3K/Akt pathway are targeted by amplification, mutation and translocation more frequently than any other pathway in cancer patients, with resultant activation of the pathway [10]. In addition, it has been suggested that activation of the PI3K/Akt pathway is required for the maintenance and viability of cancer stem cells in breast cancer, prostate cancer, and brain tumor [11-13]. Inhibition of AKT activity in cancer stem cells also resulted in sensitization of cells to therapy and increased survival of mice bearing human glioma xenografts in vivo [11-14]. Hence, the PI3K/Akt pathway can be an attractive target for cancer therapy. Inhibiting the pathway might also result in differentiation of cancer stem cells [15].

### Inhibitor of PI3K internalizes BCRP in stem cells

The PI3K/Akt pathway can modulate functions of ABC transporters through various mechanisms. According to the previous studies, inhibition of the PI3K/Akt pathway caused BCRP internalization in cells. Mogi, et al. reported a reduced SP fraction in the bone marrow of Akt1-null mice [16]. Enforced expression of Akt increased the SP fraction in the wild-type mouse bone marrow but not in the Bcrp^−/−^ bone marrow. They also showed that treatment of SP cells with 10 μM of LY294002 for 90 min promoted Bcrp translocation from the plasma membrane to the intracellular compartment. Similar results have been reported concerning glioma tumor stem-like cells [17]. However, the more detailed molecular mechanism of vesicular BCRP trafficking regulated by the PI3K/Akt pathway mostly remains to be elucidated. Alteration of subcellular BCRP localization was also observed in porcine non-SP cells, LLC-PK1 [18]. Treatment with 20 μM of LY294002 for 90 min caused BCRP internalization from the cell surface. Transfection of the dominant-negative Akt plasmid resulted in internalization of a part of BCRP in LLC-PK1 cells. Therefore, it was hypothesized that cellular distribution of BCRP might be regulated by the PI3K/Akt signaling pathway in non-stem cells as well as stem cells.

### LY294002 inhibits BCRP without affecting its translocation

**Figure 1:**
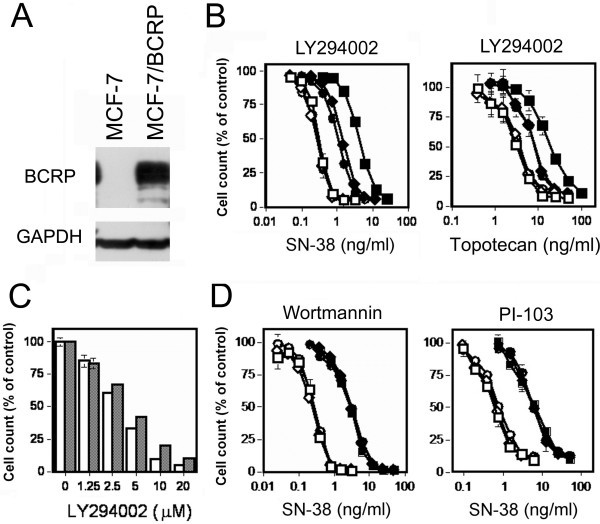
**Establishment of MCF-7/BCRP cells and effects of PI3K inhibitors on anticancer drug resistance.** (**A**) BCRP expression in MCF-7/BCRP cells. Protein sample (20 μg) was loaded in each lane and BCRP was detected by western blot analysis with the anti-BCRP antibody, BXP-21. (**B**) Effects of LY294002 on anticancer drug resistances of MCF-7/BCRP cells. Cells (3 x 10^4^/well) were seeded into 12-well plates, and cultured in the absence or presence of various concentrations of anticancer drugs with or without LY294002 for 4 days. Cell numbers were determined, and presented as percentages relative to those of control cells cultured in the absence of anticancer agents. Open symbols, MCF-7 cells. Closed symbols, MCF-7/BCRP cells. Square, without LY294002. Lozenge, 1 μM of LY294002. Circle, 2 μM of LY294002. (**C**) LY294002 resistance of MCF-7/BCRP cells. Cells (3 x 10^4^/well) were seeded into 12-well plates, and cultured in the absence or presence of various concentrations of LY294002 for 4 days. Cell numbers were determined, and presented as percentages relative to those of control cells cultured without LY294002. Open bars, MCF-7 cells. Dotted bars, the MCF-7/BCRP cells. (**D**) Effects of wortmannin or PI-103 on SN-38 resistances of MCF-7/BCRP cells. Experiment was performed in the same way as in the legend of (**B**). Open symbols, MCF-7 cells. Closed symbols, MCF-7/BCRP cells. Square, without wortmannin or PI-103. Lozenge, 1 μM of wortmannin or 50 nM of PI-103. Circle, 2 μM of wortmannin or 100 nM of PI-103. Partially reproduced by permission from Spandidos Publications. *Inc.* Imai Y, et al. (19).

**Figure 2:**
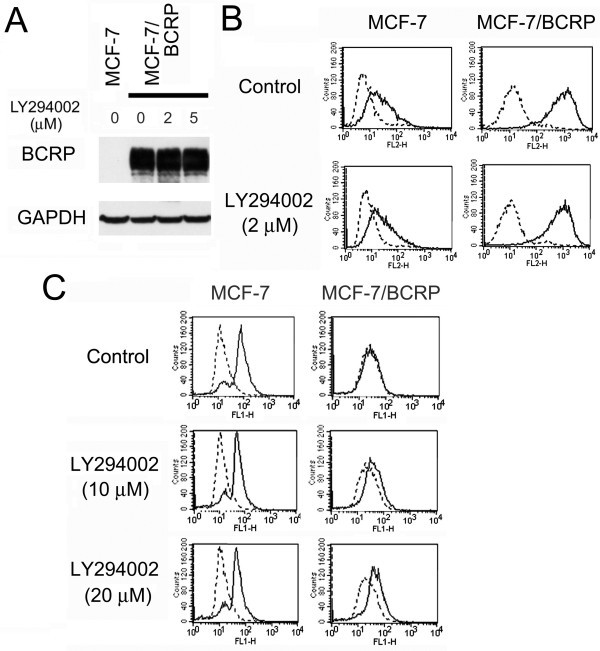
**Effects of LY294002 on BCRP expression and function.** (**A**) Effects of LY294002 on BCRP protein expression. Cells were cultured in the absence or presence of various concentrations of LY294002 for 24 h. After harvesting the cells, protein sample (20 μg) was loaded in each lane and western blot analysis was performed. (**B**) FACS analysis of BCRP expression on the cell surface. Cells were incubated for 12 h with or without 2 μM of LY294002, respectively. Trypsinized cells (5 x 10^5^–10^6^) were incubated with either biotinylated mouse IgG or anti-human ABCG2, 5D3, washed and incubated with R-phycoerythrin-conjugated streptavidin. Bold lines indicate fluorescence in the presence of 5D3 and dotted lines indicate control fluorescence in the presence of mouse IgG. (**C**) Effects of LY294002 on cellular topotecan uptake of MCF-7/BCRP cells. Trypsinized cells (5 x 10^5^) were pre-incubated with indicated concentrations of reagents for 60 min. The cells were then incubated with (solid line) or without (dotted line) 40 μM of topotecan for 30 min in the absence or presence of the reagents. Finally, cellular topotecan uptake was measured by flow cytometry. Reproduced by permission from Spandidos Publications. *Inc.* Imai Y, et al. (19).

**Figure 3:**
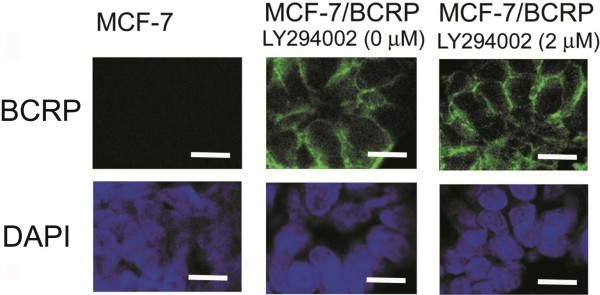
**Immunofluorescence analysis of the subcellular BCRP localization.** Cells were treated with 2 μM of LY294002 for 12 h, collected as a cell block, formalin-fixed, paraffin-embedded, and processed. BCRP was detected as green fluorescence, while cell nuclei were indicated by purple fluorescence. *Scale bar*, 30 μm. Reproduced by permission from Spandidos Publications. *Inc.* Imai Y, et al. (19).

However, effects of inhibition of the PI3K/Akt pathway in cancer cell lines were different from those in stem cells [19]. In the study, the breast cancer MCF-7 cells were transfected with exogenous *BCRP* cDNA and MCF-7/BCRP cells that stably overexpress BCRP were established. This cell line expresses epidermal growth factor receptor and may be under the control of its downstream pathways such as the Ras/MAPK/ERK pathway and the PI3K/Akt pathway. MCF-7/BCRP cells were found to overexpress BCRP (Figure 1A) and they showed significantly higher resistances to SN-38 and topotecan, which are both BCRP substrate drugs, than the corresponding parental cells (Figure 1B). MCF-7/BCRP cells also acquired slightly more resistance to the PI3K inhibitors (Figure 1C and data not shown), but the degrees of resistance did not seem to be significant considering the degrees of resistance against anticancer drugs. LY294002 significantly enhanced cytotoxicities of SN-38 and topotecan on MCF-7/BCRP cells in a dose-dependent manner, while it little affected the cytotoxicities on MCF-7 cells (Figure 1B). On the other hand, wortmannin and PI-103 did not show any significant reversing effects of BCRP-mediated drug resistances in the cells (Figure 1D and data not shown). LY294002 treatment reversed BCRP-mediated multidrug resistance, but total BCRP levels (Figure 2A) or cell surface BCRP levels (Figure 2B) in MCF-7/BCRP cells were not affected by the treatment with up to 5 or 2 μM of LY294002, respectively. Effects of LY294002 on cellular accumulation of topotecan were then evaluated by flow cytometric analysis. Cellular topotecan uptake was remarkable in the parental MCF-7 cells but not in the MCF-7/BCRP cells in the absence of LY294002 (Figure 2C). LY294002 treatment at the concentrations of 10–20 μM clearly demonstrated increased cellular topotecan uptake in a dose-dependent manner in the medium containing 40 μM of topotecan (Figure 2C). The effects were weaker than those of gefitinib but were stronger than estrone in this situation (data not shown). This surprising result suggested that trafficking problem was not a main reason for the loss of BCRP function mediated by LY294002. Fluorescence immunohistochemistry confirmed that MCF-7/BCRP cells treated with LY294002 expressed BCRP mainly on the plasma membrane in this experimental setting (Figure 3).

For the past decade, PI3K/Akt inhibitors have been known to cause more favorable outcomes when co-administered with usual anticancer drugs. Deregulation of drug transporters is one of the postulated mechanisms. It had been reported that inhibition of the PI3K/Akt pathway modulated BCRP-mediated drug transport via BCRP translocation in SP cells in the bone marrow and glioma stem-like cells. It was also reported that inhibition of the PI3K/Akt pathway resulted in BCRP translocation in renal polarized cells [16-18]. However, effects of the PI3K/Akt inhibitors on BCRP function were found to be considerably different in *BCRP*-transfected MCF-7 cells. LY294002 did not affect cellular BCRP distribution in the cancer cell line but exerted inhibitory effects on BCRP-mediated topotecan efflux. Thus, LY294002 demonstrated BCRP-inhibitory effects different from those observed in the previous studies. Then, two possible explanations were postulated to explain the results. The first one was the difference of cell characters between stem cells and non-stem cells. Regulation of subcellular BCRP distribution by the PI3K/Akt pathway might be specific to a subset of cells such as stem/stem-like cells. It is known that the PI3K/Akt signaling pathway is activated and plays a role in the maintenance of pluripotency and viability of stem cells [7]. BCRP is expressed on the cell surface of pluripotent stem cells but becomes downregulated with cell maturation. On the other hand, the PI3K/Akt signals have been reported to regulate activity and trafficking of some transporters and/or receptors, such as transferrin receptor, low-density lipoprotein receptor, the amino-acid transporter 4F2, integrin, and Glut1 through regulating endocytosis and recycling [20,21]. Therefore, the PI3K/Akt signaling pathway might be involved in mechanisms of BCRP trafficking to the cell surface and maintenance of the pluripotency of stem cells. Another explanation was that some of the BCRP protein might be internalized, but expression levels of overexpessed BCRP in cancer cells are too high to discern internalized BCRP and most of BCRP would be left uninternalized. But, it was supposed that this hypothesis could not successfully explain the data that LY294002 clearly and remarkably sensitized MCF-7/BCRP cells to BCRP-substrate drugs. Accordingly, interaction of LY294002 with the drug binding sites of BCRP was thought to explain the reversal effects. Since BCRP overexpression did not result in acquisition of comparable degrees of LY294002 resistance with those of anticancer drugs (Figure 1C), LY294002 would not be a transport substrate of BCRP but it would competitively interact with the drug binding site.

### Inhibitory effects of LY294002 on MRP1 and P-gp

LY294002 has been reported to competitively inhibit MRP1-mediated doxorubicin transport [22]. Besides, MRP1 expression has been found to be regulated by the PI3K/Akt pathway. Treatment of myelogenous leukemia cells with wortmannin resulted in lower levels of phosphorylated Akt, downregulated MRP1 expression, and decreased rho123 extrusion in dye efflux assay [23]. In another report, modulation of PI3K activity with the use of constitutively active and dominant-negative inhibitors was found to affect the ability of prostate cancer cells to respond to chemotoxic treatments [24]. MRP1 expression was upregulated as a consequence of PI3K activation in prostate cancer cells. Thus, LY294002 can also exert inhibitory effects on MRP1 function via dual mechanisms, i.e., competitive block of substrate transport and modulation of expression.

LY294002 has also been observed to antagonize transport activity of P-gp without influencing its expression and antagonize P-gp-mediated multidrug resistance [25]. Authors of this report discussed that suppression of the apoptotic pathway and regulation mechanism of the transport activity controlled by the PI3K/Akt kinase pathway might be additive to P-gp-mediated drug resistance. However, effects of other inhibitors of the pathway were not tested in the report, and we suspect that inhibition of P-gp-mediated drug transport by LY294002 might have been irrelevant to inhibition of the PI3K/Akt pathway. Taken together, LY294002 can inhibit all P-gp, MRP1 and BCRP.

### Flavonoid as seeds of cancer chemotherapeutic drugs

**Figure 4:**
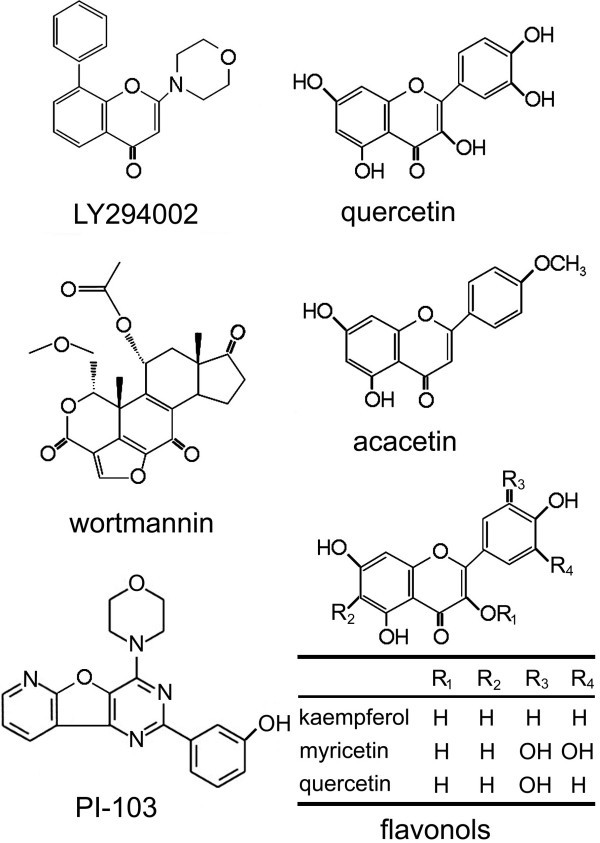
Chemical structures of the PI3K inhibitors and related flavonoids.

LY294002 was designed as a synthetic PI3K inhibitor based on quercetin, a naturally occurring flavonoid. Quercetin and other flavonoids had been found to reverse BCRP-mediated drug resistance to variable degrees in the previous study [26]. In fact, the chemical structure of LY294002 is very similar to those of quercetin and the strongest flavonoid that reverses BCRP-mediated drug resistance, acacetin. But the chemical structures of wortmannin and PI-103 are quite different (Figure 4). Flavonoids are polyphenolic compounds which are abundant in fruits, vegetables, and plant-derived beverages such as tea, green tea and wine. Flavonoids inhibit a broad range of protein kinases including PI3K [27-29]. It was reported that inhibitory activity against PI3K was noted in many flavonoids, among which flavonols (kaempferol, quercetin, myricetin, quercetagetin, et al.) showed very strong inhibitory effects [29]. These naturally occurring substances can inhibit PI3K kinase activity and function of ABC transporters by altering cellular distribution and by competitive block of substrate transport. Therefore, flavonoids and their derivatives could be a promising source of anticancer drugs.

## Conclusions

**Figure 5:**
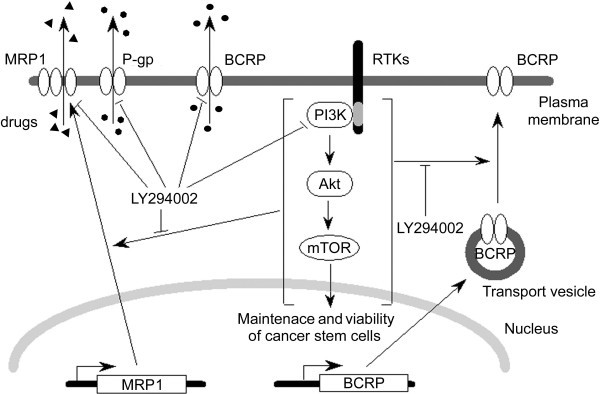
Summary of inhibitory effects of LY294002 on the PI3K/Akt signaling pathway and ABC transporters that characterize stem cells.

In addition to BCRP-internalizing effect in stem cells, LY294002 would competitively inhibit transport activity of BCRP. LY294002 also inhibits transport by P-gp and MRP1. Since LY294002 thus exerts versatile inhibitory effects on the ABC transporters that characterize stem cells and cancer stem cells (Figure 5), it can be a lead for developing more effective and tolerable reagent for cancer treatment. On the other hand, LY294002 and its derivatives may cause severe adverse effects by inhibiting many cellular signaling pathways and transporters and/or channels simultaneously. Potential side effects should be fully investigated using in-vivo models before clinical use. Furthermore, the most intriguing question that remains unresolved is why inhibiting the PI3K/Akt pathway results in internalization of BCRP in stem cells but not in non-stem cells. Elucidating this point would greatly add much on the strategy of cancer chemotherapy in the future.

## Abbreviations

ES cells: Embryonic stem cells; Rho123: Rhodamine 123; Hoechst: Hoechst 33342; SP: Side population; ABC: ATP-binding cassette; P-gp: P-glycoprotein; MDR1: Multidrug resistance 1; BCRP: Breast cancer resistance protein; MRP1: Multidrug resistance-associated protein 1; PI3K: Phosphatidylinositol-3-kinase; PIP_2_: Phosphatidylinositol 4,5-bisphosphate; PIP_3_: Phosphatidylinositol 3,4,5-triphosphate.

## Competing interests

The authors declare no competing interests regarding this manuscript.

## Authors’ contributions

YI contributed to collection of references, analysis, and drafting the manuscript. HY, YO, and YU contributed to the critical revision. All authors read and approved the final manuscript.
